# Health shock and preference instability: assessing health-state dependency of willingness-to-pay for corrective eyeglasses

**DOI:** 10.1186/s13561-019-0249-3

**Published:** 2019-11-07

**Authors:** Muhammed Nazmul Islam, Atonu Rabbani, Malabika Sarker

**Affiliations:** 1 BRAC James P Grant School of Public Health, BRAC University, 5th Floor, (Level-6), icddr,b Building, 68 Shahid Tajuddin Ahmed Sharani, Mohakhali, Dhaka, 1212 Bangladesh; 2 Department of Economics, University of Dhaka, Dhaka, 1000 Bangladesh; 3 Institute of Public Health, Heidelberg University, Im Neuenheimer Feld 130.3, Marsilius Arkaden - 6. Stock, 69120 Heidelberg, Germany

**Keywords:** State-dependent preferences, Willingness to pay, Triple-bounded dichotomous choice experiment, Refractive errors, Corrective eyeglasses, D12, D9, I1

## Abstract

**Background:**

Differences in contingent valuation (CV) estimates for identical healthcare goods can cast considerable doubt on the true economic measures of consumer preferences. Hypothetical nature of CV methods can potentially depend on the salience, context and perceived relevance of the good or service under consideration. Thus, the high demand elasticity for healthcare goods warrants careful selection of study population as the contexts of valuation significantly changes after experiencing health shock.

**Methods:**

In this study, using triple-bounded dichotomous choice (TBDC) experiments, we test how negative health shock (namely, being diagnosed with refractive errors), can alter preference over a common health good (namely, corrective eyeglasses). We compared elicited WTP of diagnosed patients with a synthetically constructed comparable cohort without the same health shock, controlling for the possible self-selection using a number of matching techniques based on the observable socio-demographic characteristics.

**Results:**

The consumers diagnosed with vision problems exhibit a rightward shift in their demand curve compared to observationally identical consumers without such problems resulting in about 17% higher consumer surplus. The consumers without the health shock are willing to pay about BDT 762.4 [95% CI: BDT 709.9 - BDT 814.9] for corrective eyeglasses, which gets 15–30% higher for the matched with-health-shock consumers. Multivariable analyses suggest more educated and wealthier individuals are willing to pay respectively BDT 208 and BDT 119 more for corrective eyeglasses. We have tested the models for different matching protocols. Our results are fairly robust to alternate specifications and various matching techniques.

**Conclusion:**

The preferences for healthcare goods, such as eyeglasses, can significantly depend upon the respondent being diagnosed with refractive errors. Our findings have implications for general cost-benefit analyses often relying on WTP, which can vary depending on the contexts. There are also increasing interests in cost recovery models, which require understanding the demand for healthcare goods and services. We find eliciting the demand needs to consider the health status of the population from which the respondents are sampled.

## Introduction

Measuring potential benefits from health interventions can be difficult. However, such estimates are essential for cost-benefit analyses (CBA) to evaluate and assess health interventions [1, 2]. Public sector agencies and development practitioners often use CBA to prioritize and select programs, projects, and policies. There are also greater emphases in recent time on cost recovery and revenue generation, where understanding consumer demand is necessary [3, 4]. Cost recovery requires revenue generations, which in turn relies on understanding demand for new or existing health products and services among the potential beneficiaries.

Willingness to pay (WTP) is a popular and frequently used tool to understand potential consumer benefits of health products and services [5, 6]. WTP is a basic building block in consumer demand and deeply rooted in standard consumer theory. Individual or aggregate WTP for a product or service traces out the consumer demand curve. Social programs often aim to maximize benefit, measured by WTP, in relation to the program cost and attain the most favorable benefit-cost ratio, rationalizing resource allocated to the program. Knowledge of WTP can also guide how to price a product or service to maximize take-up among the targeted beneficiaries, which remains a challenge as consumers usually reveal very high demand elasticity for most health products [7]. Understanding consumers’ WTP and the demand can potentially guide the optimal pricing and subsidy policies to maximize social welfare [8–10].

Eliciting WTP and linking it with the actual preference for a new health product or service, however, can be challenging [11, 12]. Consumers may have no or limited prior exposure to the product rendering the perceived benefits elusive [13, 14]. The benefits may be diffused over a large number of beneficiaries, which is often true for public goods such as environment protection [15, 16]. Even for familiar private goods, the perceived health benefits may well be dependent on realized health states and agent’s preference, hence, WTP, consumer demand, and choices may not be static in nature, as commonly assumed in standard economic theory [17, 18].

In this paper, we test how WTP for a certain health product, eyeglasses for correcting common refractive errors, can vary with and without a particular health shock, namely, being diagnosed with refractive errors and prescribed by the doctor to use corrective eyeglasses. Refractive error is a condition in which patients experience blurry vision because of their distorted eye shapes not allowing the light to bend correctly for a proper focus [19]. Most common refractive error conditions include myopia, hyperopia, presbyopia, and astigmatism. These conditions are commonly corrected with eyeglasses, an otherwise cost-effective intervention [20].

Globally, about 253 million people around the world have impaired vision and the uncorrected refractive error is the leading cause of visual impairment [21]. Such conditions adversely affect the day-to-day functioning of the patients and the global economic productivity loss due to uncorrected refractive error is estimated to be US$ 268.8 billion [22]. About 90% visually impaired people live in the low- and middle-income countries (LMICs) such as Bangladesh [23]. According to the last national blindness and low vision survey, about 4.6 million Bangladeshi citizens were estimated to suffer from different refractive error conditions [24]. However, developing countries lack in terms of awareness, financial, and human resources to deliver eye-care services adequately [25]. In such resource-poor settings, understanding the demand and WTP for corrective eyeglasses can help design equitable and affordable eye-care products and services [26].

Understanding WTP for eyeglasses is particularly useful because they are otherwise familiar healthcare products. We assess the perceived benefits from using corrective eyeglasses through eliciting WTP in a common contingent valuation (CV) framework, namely triple bounded dichotomous choice [27, 28]. We quasi-experimentally simulate the absence and presence of health shocks of being diagnosed with refractive errors by synthetically constructing two groups using a number of different matching techniques [29, 30]. Hence, we construct two observationally equivalent groups, one is diagnosed with the vision problems, while the other is not. We elicit WTP for corrective eyeglasses from both with- and without-health shock groups and estimate how differently they value vision correction through eyeglasses based on their realized health states. The remainder of the paper is organized as follows. The methods are described in section 2, whereas findings are presented in section 3. Section 4 includes the discussion and section 5 concludes.

## Methods

### Context of the study

We use data from a study aiming to understand eye related healthcare needs and care-seeking behaviors among people living in low-income communities or “slums” in Dhaka. We primarily focus on common refractive errors which can be treated with lenses (eyeglasses or rarely used contact lenses). Eyeglasses are considered very cost-effective solutions to refractive errors [31, 32]. Typically, corrective eyeglasses are categorized into two types: (a) custom-made spectacles, and (b) ready-made spectacles. Both can potentially improve visual acuity for people with refractive errors. However, ready-made spectacles are generally more cost-effective [33, 34]. Nevertheless, owing to perception and competing financial needs, low-income consumers often do not prioritize using eyeglasses [32, 35]. Hence, understanding the demand for an otherwise familiar private healthcare product such as eyeglasses or spectacles provides an interesting setting to test the effects of health shocks on elicited WTP.

### Sampling

We carry out the data collection in three distinct phases. In the first phase, we collect data from four low-income communities located in Dhaka City Corporation. All households in these areas are registered by a community health outreach program of a prominent local NGO. We use the household rosters as sampling frames and randomly select 400 households from each of the four selected areas. We then randomly select an adult member (i.e. aged 18 years or above) from each of the selected households as survey respondent if s/he agrees to provide a written consent. We collect personal and household information from each of the participants. Additionally, we elicit WTP for corrective eyeglasses or spectacles using triple bounded dichotomous choice experiments.

In the second phase, we interview patients with diagnosed refractive error conditions. We choose five specialized eye-care hospitals in Dhaka based on formative research carried out in the same four communities. Sampling is based on a systematic random technique where we choose the first respondent randomly every day and then, based on the sampling interval of the facility, we interview every fourth to sixth exiting patients. For a specific hospital the sampling interval was constant and set based on its average daily patient flow. We carry out the survey for 22 consecutive working days in a month to ensure as much representations as possible. We interview 558 patients and collect the personal and household information using the same survey instruments. Just like the first phase, we elicit WTP for eyeglasses using the same CV method. For the minors (i.e. aged below 18 years), we interview their accompanying guardians.

In the third and final phase, to compare hypothetical WTPs with the market price, we interview 361 shoppers and collect information on prices they have paid for their corrective eyeglasses. We use similar systematic random sampling techniques to choose shoppers from three optic shops and conducted interviews for 10 consecutive business days.

### Willingness-to-pay elicitation

We conduct triple bounded dichotomous choice (TBDC) experiments to elicit WTP for corrective eyeglasses. TBDC is a popular CV technique, where a series of dichotomous choice questions, accept or reject a bid, are asked that progressively narrow down the WTP bounds [27, 36, 37]. The initial bid is chosen randomly from three values based on formative research to control for the anchoring biases [38, 39].

**Fig. 1:**
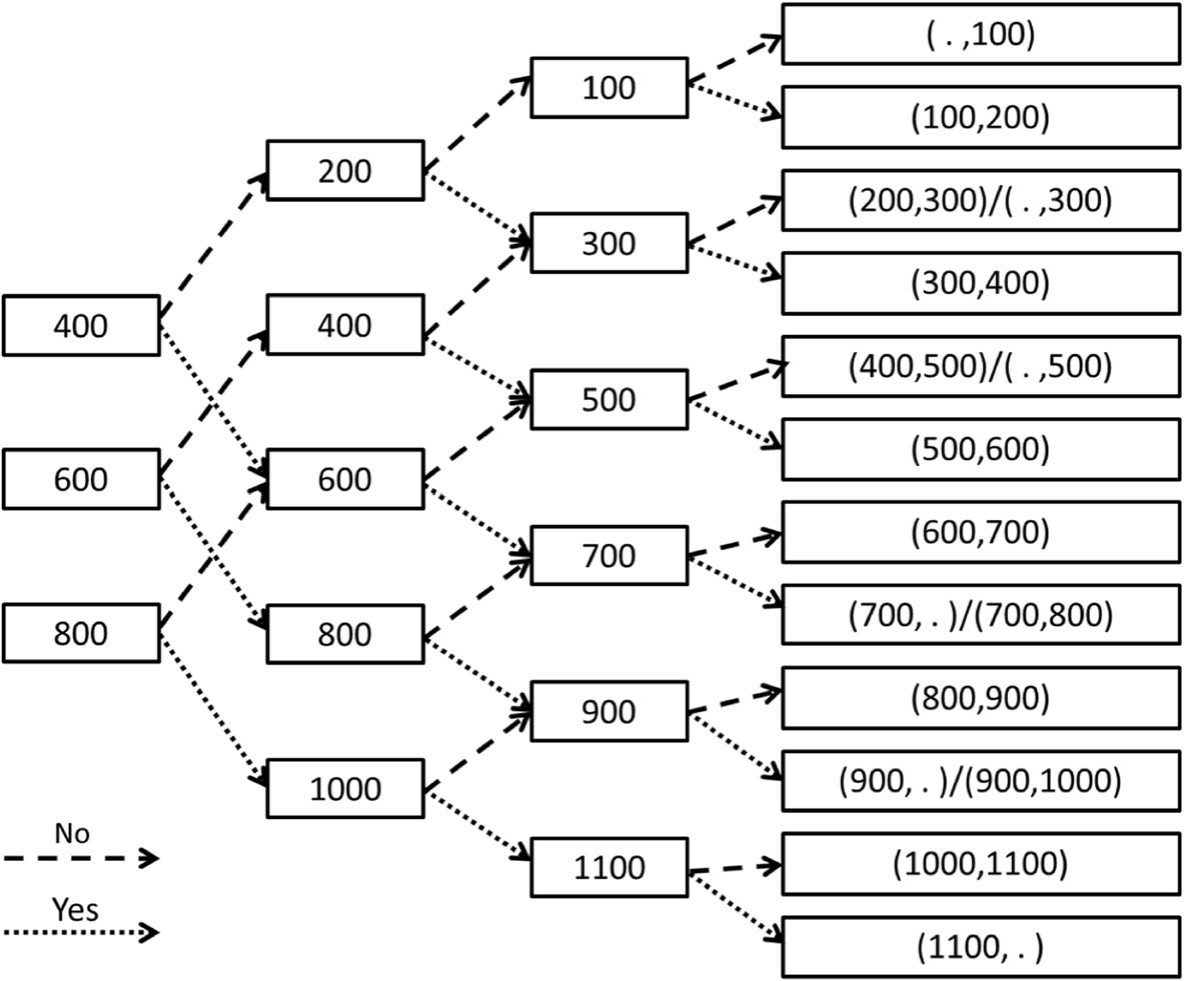
Schematic Representation of the Choice Experiments. Note: Individuals (558 diagnosed with refractive errors and 1560 not-diagnosed) were randomly selected to elicit WTP for refraction corrective eyeglasses. They were asked whether they would pay the amount (stated in each node) to purchase corrective eyeglasses. The second consecutive bids were increased (decreased) based on the acceptance (rejection) of the random initial bid. Final intervals of the respondent’s WTP contain both left- and right-censored (e.g. [., 100] and [1100, .] respectively) and interval-coded observations (e.g. [500, 600])

The subsequent bids are based on accepting or rejecting the initial and subsequent bids (see Fig. 1). Following the sequence of interlinked bidding questions, all participants reach a WTP interval of BDT 100. However, accepting or rejecting all of the three bids lead to a censored interval. Rejecting all three bids gives us a left-censored interval (e.g. [.,100]). Hence, the WTP is assumed to be less than the upper bound. On the other hand, accepting all three sequential bids gives us a right censored interval and we get only a lower bound of the consumers’ WTP.

Identical choice experiments are conducted for both groups: with and without refractive health shocks. Participants of the without-health shock group are not diagnosed with any refractive error condition. Thus, prior to conducting TBDC experiment, we condition them in order to provide cognitive resemblances of refractive errors. We randomly ask each of the participants in the without-health shock group to wear +1D or + 2D powered eyeglasses. Powered glasses make their vision blurry and then we replace it with zero-powered glasses. However, no mental conditioning is required for the with-health shock or the patient group as they are already exposed to the health shock (i.e. refractive errors).

### Matching methods

Obviously, a simple mean comparison between the two groups for whom we elicit WTP will not allow us to identify the impacts of health shocks on WTP for eyeglasses. The two populations are not the same and respondents have self-selected to visit ophthalmologists to seek eye care services. To reduce the observed differences between these two groups, we apply propensity score matching (PSM) techniques to create a balanced counterfactual for the group with health shock based on socioeconomic features [29, 30, 40].

We have applied five different matching techniques: one-to-one nearest neighborhood matching with and without replacement, kernel matching, radius matching, and Mahalanobis metric matching. Based on the matching techniques, we form five groups each containing 558 individuals from the without-health-shock group. Moreover, to find the best counterfactual from these five matched sub-samples, we assess each of them by different balance checking criteria [41]. According to the assessment of these criteria, we find that, among the five alternative groups of counterfactuals, the one-to-one nearest neighborhood with replacement matching technique generates the best-matched samples (see Additional file 1: Table S1). However, we report results from all five methods to check the robustness of our findings.

### Statistical analysis

Interval regression methods are used to empirically estimate the effects of health shock (i.e. diagnosed with refractive errors) on the preferences of corrective eyeglasses. We assume that true WTP (${Y}_i^{\ast }$) of the consumer is latent and takes the following linear functional form

$$ {\mathrm{Y}}_{\mathrm{i}}^{\ast }={\mathrm{X}}_{\mathrm{i}}\upbeta +{\mathrm{H}}_{\mathrm{i}}\upgamma +{\upvarepsilon}_{\mathrm{i}} $$

where, X_i_ is a vector of observable socioeconomic characteristics (i.e. age, gender, education, occupation, family income, asset ownership, etc.) that we control for to estimate the effects of health-shock (*H*_*i*_). *H*_*i*_ is a binary variable, where *H*_*i*_ = 1 if the individual is diagnosed with refractive errors and *H*_*i*_ = 0 otherwise. We assume that conditional on the socioeconomic characteristics, the event of health-shock is independent of the WTP for corrective eyeglasses (unconfoundedness assumption, see [42]).

However, triple-bounded dichotomous choice (TBDC) experiment allows us to restrict the latent WTP within an interval, or ${Y}_i^{\ast}\in \left[{Y}_i^l,{Y}_i^h\right)$. By randomly varying the initial bid across individuals, responses are found into sixteen (16) intervals, including six (6) censored intervals (see Fig. 1 for details). Assuming standard normal disturbance or [*ε*_*i*_| ***X***_***i***_, *γ*_*i*_)~*N*(0, 1)], we can model the probability of lying within an interval or $\Pr \left({Y}_i^l\le {\boldsymbol{X}}_{\boldsymbol{i}}\boldsymbol{\beta} +{H}_i\gamma <{Y}_i^h\right)$ using standard normal cumulative function. Quantitative nature of our dependent variable (i.e. WTP for corrective eyeglasses) and intervals with known cut-points (i.e. ${Y}_i^l,{Y}_i^h$) allow us to estimate our model using interval regression techniques. Interval regression method follows the ordered-probit technique (with known cut-points) and estimate the model parameters (i.e. ***β,****γ*) by maximizing the following log-likelihood function [43].

$$ \ell \left(a,\beta \right)=\sum \limits_{i=1}^N\left[{C}_i^l\log \left\{\varPhi \left(\frac{Y_1^l-{X}_i\beta -{H}_i\gamma }{\sigma}\right)\right\}+\left(1-{C}_i^l-{C}_i^R\right)\log \left\{\varPhi \left(\frac{Y_2^h-{X}_i\beta -{H}_i\gamma }{\sigma}\right)-\varPhi \left(\frac{Y_1^l-{X}_i\beta -{H}_i\gamma }{\sigma}\right)\right\}+\dots +\left(1-{C}_i^l-{C}_i^R\right)\log \left\{\varPhi \left(\frac{Y_J^h-{X}_i\beta -{H}_i\gamma }{\sigma}\right)-\varPhi \left(\frac{Y_{J-1}^l-{X}_i\beta -{H}_i\gamma }{\sigma}\right)\right\}+{C}_i^R\log \left\{\varPhi \left(\frac{Y_J^l-{X}_i\beta -{H}_i\gamma }{\sigma}\right)\right\}\right] $$

where, *Y*_*i*_ = [*Y*_1_ < *Y*_2_ < *Y*_3_ < … < *Y*_*J*_] are the interval cut-points and *Y*_*i*_ = 0 for censored observations; ${C}_i^l=1$ if *Y*_*i*_ = 0, otherwise ${C}_i^l=0$ and ${C}_i^R=1$ if *Y*_*i*_ = *Y*_*J*_, otherwise ${C}_i^R=0$. Due to strong assumption that ${Y}_i^{\ast }$ (conditional on X_*i*_, *H*_*i*_) satisfies the classical linear regression model (CLRM) assumptions. The coefficients (i.e. ***β***, *γ*) are interpretable as the coefficients of the ordinary least squares (OLS).

In this paper, we conduct empirical estimations into two phases. In the first phase, we estimate the mean WTP for corrective eyeglasses by fitting constant-only models for unmatched and all the five matched cohorts. As the observed WTP are within intervals, predicted mean WTP is attained by iteratively maximizing the log-likelihood of the data given a mean predicted value. Later, we introduce health-shock in the model and estimate the differences in mean WTP between the with- and without-health-shock groups.

In the second phase, instead of fitting the full model on different matched cohorts, we concentrate our analyses on the best statistically representative matched cohort (i.e. One-to-One Nearest Neighborhood matching technique with replacement). We fit the full model to estimate the effects of health-shock on WTP for corrective eyeglasses controlling for socioeconomic variations within the matched cohort. However, we also apply regression-adjusted matching technique to estimate the health-shock effect. First, we conduct propensity score matching and use the propensity scores as weights to fit the full model using all 2118 observations [44–46]. All analyses were carried out in Stata® version 13.1.

## Results

### Summary statistics

We first report the differences in observable characteristics between the groups with and without health shocks and also assess the comparability of the two groups. Table 1 reports descriptive statistics of the unmatched samples, whereas, Tables 2 and A2 (see Additional file 1) report the same for the different matched sub-samples. We also report *p*-values to test the mean differences between the two groups.

**Table 1:** Summary Statistics – Unmatched Samples

	[1]	[2]	[3]	[4]	[5]
	Entire Sample [*N* = 2118]		Unmatched Without-Health-Shock Group [*N* = 1560]	With-Health-Shock Group [*N* = 558]	*P* Value
	Mean	SD			
Female	57.0%	49.5%	58.7%	52.3%	0.009 ***
Married	80.1%	39.9%	84.0%	69.4%	0.000 ***
Owns a TV	74.7%	43.5%	72.4%	81.0%	0.000 ***
Owns a mobile	85.5%	35.2%	81.7%	96.2%	0.000 ***
Respondent’s Contribution to Family Income	38.1%	41.1%	39.3%	34.6%	0.021 **
Family Income (BDT)	16,631.3	15,591.1	14,968.0	21,281.7	0.000 ***
Land (Decimal)	24.89	128.86	11.0	63.73	0.000 ***
Age	35.9	14.1	34.8	38.6	0.000 ***
Respondent’s Income (BDT)	5614.1	7897.4	5375.6	6280.6	0.020 **
Family Size					
1–2	13.7%		14.8%	10.6%	0.000 ***
3–4	50.5%		53.2%	43.0%	
5+	35.8%		32.0%	46.4%	
Occupations					
Wage Workers	14.9%		19.2%	2.9%	0.000 ***
Self-employed	10.0%		9.9%	10.4%	
Garment Workers	8.6%		10.5%	3.4%	
Service	11.8%		8.5%	20.8%	
Homemakers	33.8%		33.9%	33.7%	
Others	20.9%		18.0%	28.8%	
Education					
Never been to School	35.6%		39.5%	24.7%	0.000 ***
Primary	27.6%		31.2%	17.6%	
Secondary	17.0%		17.9%	14.3%	
SSC/Dakhil/Equivalent	8.1%		5.9%	14.5%	
HSC/Alim/Equivalent	5.6%		3.2%	12.2%	
Graduate	3.6%		1.8%	8.6%	
Post Graduate	1.5%		0.4%	4.3%	
Others	1.0%		0.1%	3.8%	

(a) *P* value for comparison between With and Without-Health-Shock groups: generated using t-test of difference in means for continuous variables and Chi-square test of independence for categorical variables, (b) Asterisks indicate statistical significance (*** *p* < 0.01, ** *p* < 0.05), (a) US$ 1 is equivalent to BDT 84.397

**Table 2:** Summary Statistics – Matched Sub-Samples

	[1]	[2]	[3]	[4]	[5]
	With-Health Shock Group [*N* = 558]	Without-Health-Shock Group: 1 to 1 Matching (Replaced) [*N* = 558]	*P* Value	Without-Health-Shock Group: 1 to 1 Matching (Not Replaced) [*N* = 558]	*P* Value
Female	52.3%	53.2%	0.765	61.8%	0.001 ***
Married	69.4%	63.6%	0.042 **	74.9%	0.039 **
Owns a TV	81.0%	82.8%	0.437	81.4%	0.878
Owns a mobile	96.2%	94.3%	0.122	95.3%	0.457
Respondent’s Contribution to Family Income	34.6%	35.2%	0.827	31.0%	0.160
Family Income (BDT)	21,281.7	19,948.7	0.283	17,808.9	0.003 ***
Land (Decimal)	63.73	44.59	0.081	20.73	0.000 ***
Age	38.6	40.9	0.023 **	38.7	0.928
Respondent’s Income (BDT)	6280.6	6604.0	0.626	5177.8	0.060 *
Family Size					
1–2	10.6%	10.9%	0.156	10.6%	0.982
3–4	43.0%	37.5%		42.5%	
5+	46.4%	51.6%		46.9%	
Occupations					
Wage Workers	2.9%	3.6%	0.998	3.2%	0.004 ***
Self-employed	10.4%	10.6%		11.7%	
Garment Workers	3.4%	3.2%		3.1%	
Service	20.8%	20.8%		15.6%	
Homemakers	33.7%	32.6%		44.0%	
Others	28.8%	29.2%		22.4%	
Education					
Never been to School	24.7%	32.2%	0.164	31.4%	0.000 ***
Primary	17.6%	15.1%		20.6%	
Secondary	14.3%	15.4%		19.1%	
SSC/Dakhil/Equivalent	14.5%	14.2%		13.8%	
HSC/Fazil/Equivalent	12.2%	9.5%		8.4%	
Graduate	8.6%	7.0%		5.2%	
Post Graduate	4.3%	3.4%		1.3%	
Others	3.8%	3.2%		0.2%	

(a) *P* value for comparison between With-Health-Shock group and matched sub-samples of Without-Health-Shock group: generated using t-test of difference in means for continuous variables or Chi-square test of independence for categorical variables, (b) Asterisks indicate statistical significance (*** *p* < 0.01, ** *p* < 0.05, * *p* < 0.1), (a) US$ 1 is equivalent to BDT 84.397

In the pooled sample of 2118 respondents, 57% of the respondents are female with an average age of 36 years. Around 80% of them are married with an average family size of 4.1 (with an SD of 1.7). The average monthly family income is about 16.6 thousand BDT, equivalent to US$ 197 (with an SD of 15.5 thousand BDT or US$ 185). The respondent contributes on average 38% of the total monthly family income. Nearly 75% of the respondents have access to a television and about 86% have access to a mobile phone. Average household land ownership is about 25 decimals of land (with an SD of 129 decimals). Most respondents have below primary level education with 36% reporting no formal education and 28% with an education level of primary level or less.

We find that the groups with and without the negative health shock are observationally different in terms of different socioeconomic features. The with-health shock group has about 52% female. The group with the shock is older compared to the without-shock group with a mean age of 39 years and an SD of about 16.6 years, also more educated and has more white-collar jobs. The households in this group have a higher income and own more land assets on average compared to the without-health shock group (see Table 1).

Among the different matched counterfactuals, the one-to-one nearest neighborhood with replacement matching technique exhibits the lowest mean differences between with- and without-health shock groups (see Table 2, columns 1, 2 and 3, to compare with other methods, see Additional file 1: Table S2). Except for respondents’ ages and marital statuses, matched samples are statistically comparable based on observable socioeconomic characteristics. Two groups are similar in terms of education and occupation. Both groups have about 52% female respondents and in a month their households earn on an average BDT 20 thousand. The average land ownership of with- and without-health shock groups are respectively about 45 and 64 decimals of land. However, about half of the respondents live in a family of more than 5 members and the respondents on average contribute about 35% in their total household income. Most of the respondents have access to a television (around 80%) and a mobile phone (around 95%).

### Demand for eyeglasses at different health states

**Fig. 2:**
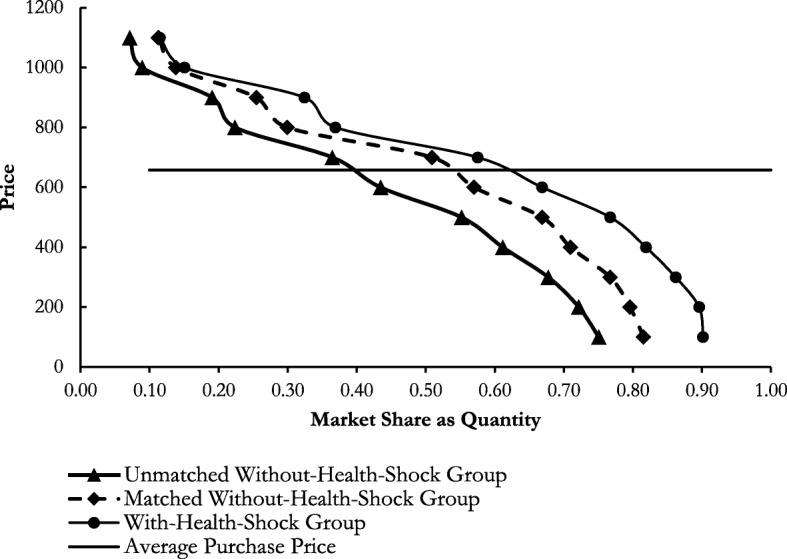
Estimated Demand Curves for Corrective Eyeglasses. Source: Authors’ calculations using triple-bounded dichotomous choice contingent valuation experiment

We draw demand curves for corrective eyeglasses using outcomes from our TBDC experiments among the with- and without-health shock groups (see Fig. 2). To derive the curves, we determine the fraction of acceptance against the lower bound (i.e. minimum price) for each interval. However, we ignore the left-censored intervals (e.g. [.,100]) to control for uncertainty in finding the lower bounds of these intervals. For all the accepted bids, we count positive responses for other lower bids. For example, a person who accepted the offer at BDT 1100 is also assumed to accept the offer for anything lower than BDT 1100. Figure 2 illustrates the estimated demand curves for corrective eyeglasses and how it varies by different health states. We also compare the demands against the average purchase price of BDT 657 from the 361 random shoppers.

Among the without-health shock group before matching, at the lowest price point of BDT 100, 75% of the respondents are willing to purchase corrective eyeglasses. The demand for corrective eyeglasses drops by nearly half for the average purchase price of BDT 657 and only 7% of the respondents are willing to purchase at the price of BDT 1100.

We find a rightward shift in demand for the with-health shock group (see Fig. 2). Among the with-health shock group, at the lowest price point of BDT 100, 90% of the respondents are willing to purchase eyeglasses. It drops to approximately 63% if price increases to BDT 657 and further drops to 11% if price increases to BDT 1100. Interestingly, even at this highest price, demand is 4 percentage points higher compared to the without-health shock group.

Figure 2 also shows that the matching mitigates the rightward shift in demand. Among the matched without-health shock group, 82% of the respondents are willing to purchase eyeglasses at BDT 100. Demand drops to approximately 55% at the price of BDT 657. Moreover, it remains at 11% (similar to with-health shock state) if price increases to BDT 1100. However, in spite of the reduced differences from matching, with-health shock demand for corrective eyeglasses remains higher compared to that of the without-health shock group.

### Consumer surplus attained at different health states

**Fig. 3:**
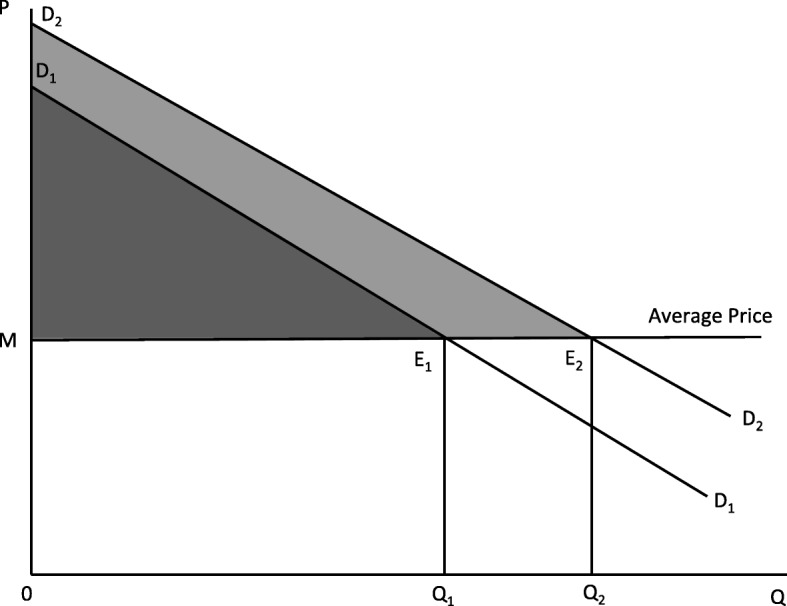
The Basic Demand Model and Suggested Consumer Surplus. Source: Authors’ rendition

We assume a constant marginal cost or an infinitely elastic market supply at the estimated average purchase price of BDT 657 for corrective eyeglasses. We also estimate linear demand functions for each of the demand curves presented in Fig. 2 and corresponding consumer surpluses at different health shock states (as we have seen in Fig. 3). Table 3 reports consumer surpluses attained for the average purchase price point of BDT 657 for the different demand functions.

**Table 3:** Cost and Consumer Surplus Simulated from the Suggested Demand Curves

Case	[1]	[2]	[3]	[4]
	Slope	Intercept	Consumer Surplus	Cost
Unmatched Without-Health-Shock Group	− 1292.7	1150.8	94.1	250.9
Matched Without-Health-Shock Group	− 1211.0	1220.9	131.1	305.9
With-Health-Shock Group	− 1077.3	1231.5	153.0	350.3

(a) Slopes and intercepts were estimated by fitting linear regressions for each of the demand curves demonstrated in Fig. 2, (b) Consumer surplus is obtained with an assumption of market equilibrium at the average purchasing price of BDT 657, (c) Column 4 of this table shows the average cost incurred at equilibrium conditions with the three different demand curves presented in Fig. 2, (d) Fig. 3 graphically demonstrates the consumer surplus calculation

A consumer representing the unmatched without-health shock group attains a surplus of about BDT 94. However, after being diagnosed with refractive error, a respondent accrues a consumer surplus of BDT 153 which is BDT 59 or about 63% higher compared to the group without the health shock. Minimizing between-group differences through matching reduces the difference in consumer surplus between the two groups. Consumer surplus attained by the matched without-health shock group is about BDT 131 suggesting an additional consumer surplus of BDT 22 or 17% for a vision correction through eyeglasses.

### Average willingness-to-pay of the with- and without-health shock groups

We estimate average WTP for corrective eyeglasses using interval regression models and report the results in Table 4. For entire unmatched samples, the average WTP for corrective eyeglasses is about BDT 665 (95% CI: 642.5–686.8). The health shock (i.e., diagnosed with refractive errors) results in about BDT 258 (95% CI: 208.0–307.7) or 43.2% higher WTP for the diagnosed patients compared to the without-health shock group (see columns 2 and 5, Table 4). However, for a cohort from the without-health shock group matched with the with-health shock group (using one-to-one nearest neighborhood match with replacement) suggests an average WTP of about BDT 809 (95% CI: 774.7–843.0). After matching, health shock results in about BDT 117 (95% CI: 52.3–181.4) or 15.3% higher WTP compared to the matched cohort from the without-health-shock group.

**Table 4:** Willingness-to-Pay for Corrective Eyeglasses

	[1]	[2]	[3]	[4]	[5]
	Average WTP	Shock induced changes in WTP	Standard Error	95% Confidence Interval	Shock as a percentage of Average WTP
Entire Sample	664.6 ***	–	11.32	642.5–686.8	–
Unmatched Without-Health-Shock Group	596.8 ***	–	12.86	571.6–622.1	–
= 1 if diagnosed with refractive errors	–	257.8 ***	25.44	208.0–307.7	43.20%
1 to 1 Matched (Replaced) Sub-Sample	808.9 ***	–	17.41	774.7–843.0	–
Matched Without-Health-Shock Group	762.4 ***	–	26.78	709.9–814.9	–
= 1 if diagnosed with refractive errors	–	116.8 ***	32.93	52.3–181.4	15.32%
1 to 1 Matched (Not Replaced) Sub-Sample	773.9 ***	–	16.45	741.7–806.2	–
Matched Without-Health-Shock Group	691.9 ***	–	23.62	645.6–738.2	–
= 1 if diagnosed with refractive errors	–	171.3 ***	31.37	109.8–232.8	24.76%
Kernel Matched Sub-Sample	772.0 ***	–	16.49	739.6–804.3	–
Matched Without-Health-Shock Group	687.4 ***	–	23.70	640.9–733.8	–
= 1 if diagnosed with refractive errors	–	176.0 ***	31.43	114.4–237.6	25.60%
Radius Matched Sub-Sample	757.8 ***	–	16.15	726.1–789.4	–
Matched Without-Health-Shock Group	659.9 ***	–	22.76	615.2–704.5	–
= 1 if diagnosed with refractive errors	–	199.0 ***	31.02	138.2–259.8	30.16%
Mahalanobis Metric Matched Sub-Sample	771.4 ***	–	15.86	740.3–802.5	–
Matched Without-Health-Shock Group	690.0 ***	–	22.14	646.5–733.3	–
= 1 if diagnosed with refractive errors	–	165.1 ***	30.33	105.6–224.5	23.93%
Weighted by Propensity Scores	792.8 ***	–	45.82	758.9–826.7	
= 1 if diagnosed with refractive errors	–	234.6 ***	31.52	172.9–296.4	29.59%

(a) *Average WTP* - is the mean predicted value obtained through constant-only model where the log-likelihood of the model is iteratively maximized given a mean predicted value, (b) *Shock induced changes in WTP* - is the marginal WTP attributable to the health shock (i.e. being diagnosed with refractive errors); it is the coefficient obtained through fitting interval regression model with a dummy variable which is equals to 1 for experiencing the health shock and 0 otherwise, (c) Each of the sub-samples (i.e. obtained using the respective matching techniques) contains 1116 matched respondents of With- and Without-Health-Shock groups, (d) The *Matched Without-Health-Shock Group* contains 558 respondents selected as a viable match for the 558 With-Health-Shock group respondents, (e) *Shock as a percentage of Average WTP* - is obtained by calculating the *Shock induced changes in WTP* as the percentage of the *Average WTP* of the entire/sub-samples; it describes by how much the average WTP for eyeglasses of the With-Health-Shock group is higher compared to that of the Without-Health-Shock group, (f) Asterisks indicate statistical significance (*** *p* < 0.01)

The WTP for corrective eyeglasses among the diagnosed with-health shock group remains higher for other matching protocols. For all the other cohorts constructed using different matching techniques, a with-health shock respondent exhibits WTP of about 24–30% higher compared to any without-health-shock respondent (see columns 2 and 5, Table 4).

However, these matching techniques can cause loss of information as we choose only 558 best-matched observations from a group of 1560 without-health shock respondents to construct a cohort of counterfactuals of the with-health-shock respondents. Hence, we use regression adjusted matching technique, where we weigh all 2118 observations based on their respective propensity scores to estimate WTP for corrective eyeglasses (see section 2.5). For regression adjusted matching method, we find that the effect of health-shock is more pronounced as a with-health-shock respondent exhibits about BDT 235 (95% CI: 172.9–296.4) or 30% higher WTP compared to the without-health shock group (see Table 4).

### Multivariable analyses: effects of health shock on healthcare preferences

Table 5 shows interval regression results of the full model described in section 2.4 and 2.5. We primarily focus on the results of the matched cohort using one-to-one nearest neighborhood with replacement (column 4, Table 5). However, we estimate models for unmatched and weighted observations as well. For the matched cohorts, we control for observed socioeconomic characteristics and estimate the marginal WTP for corrective eyeglasses associated with the health shocks.

**Table 5:** Regression Results – Effect of Health-Shock on WTP for Corrective Eyeglasses

	[1]	[2]	[3]	[4]	[5]
	Unmatched			1 to 1 (Replaced) Matched Sub-Sample	Weighted by Propensity Scores
	Without-Health Shock	With-Health Shock	Entire Sample		Entire Sample
Age	−10.14**	−6.49	−9.37***	−2.36	−8.54**
	(0.02)	(0.33)	(0.01)	(0.59)	(0.03)
Age Squared	0.06	0.04	0.06	−0.01	0.06
	(0.23)	(0.55)	(0.13)	(0.87)	(0.20)
=1 if Female	−153.20***	−15.78	−112.41***	−26.43	−84.68**
	(0.00)	(0.79)	(0.00)	(0.56)	(0.05)
=1 if Married	0.99	−58.11	−9.17	−13.89	−64.51*
	(0.98)	(0.29)	(0.75)	(0.72)	(0.07)
Education					
None	Base	Base	Base	Base	Base
Primary	3.26	79.61	18.54	53.11	38.44
	(0.91)	(0.18)	(0.46)	(0.23)	(0.25)
Above Primary	151.51***	158.14***	148.39***	208.18***	191.36***
	(0.00)	(0.00)	(0.00)	(0.00)	(0.00)
=1 if HH has TV	61.58**	78.23	61.56***	50.37	113.41***
	(0.02)	(0.12)	(0.01)	(0.18)	(0.00)
=1 if HH has Mobile Phone	172.68***	99.11	173.58***	284.30***	205.18***
	(0.00)	(0.35)	(0.00)	(0.00)	(0.00)
Land Owned (Decimal, Standardized)	57.87***	43.84	56.99***	44.73***	44.20**
	(0.00)	(0.10)	(0.00)	(0.00)	(0.01)
HH Income (BDT, Standardized)	132.31***	81.40***	113.86***	118.61***	84.73**
	(0.00)	(0.00)	(0.00)	(0.00)	(0.02)
Respondent’s Contribution to HH Income (%)	8.37	91.18	34.35	85.24	60.28
	(0.87)	(0.21)	(0.40)	(0.14)	(0.31)
Family Size					
1–2 Member(s)	Base	Base	Base	Base	Base
3–4	5.02	47.41	13.70	−87.16*	−48.73
	(0.88)	(0.47)	(0.65)	(0.08)	(0.21)
5 or More	25.21	52.48	35.04	−29.31	−24.31
	(0.51)	(0.42)	(0.28)	(0.56)	(0.58)
Occupation					
Wage Worker	Base	Base	Base	Base	Base
Self Employed	183.66***	229.41*	180.21***	241.20***	201.79***
	(0.00)	(0.06)	(0.00)	(0.01)	(0.00)
Garment Worker	246.48***	238.82	235.74***	87.57	205.33***
	(0.00)	(0.12)	(0.00)	(0.43)	(0.00)
Service	139.12***	135.33	112.01***	59.39	115.27**
	(0.00)	(0.24)	(0.01)	(0.48)	(0.02)
Homemaker	160.30***	197.98	157.07***	160.76*	177.64***
	(0.00)	(0.13)	(0.00)	(0.09)	(0.01)
Others	77.94*	139.08	71.29*	110.65	68.26
	(0.06)	(0.25)	(0.06)	(0.21)	(0.19)
Starting Bid					
BDT. 400	Base	Base	Base	Base	Base
BDT. 600	21.56	81.05*	34.87	70.01**	39.28
	(0.43)	(0.08)	(0.14)	(0.05)	(0.20)
BDT. 800	40.97	93.54**	54.05**	61.77*	57.09*
	(0.14)	(0.05)	(0.02)	(0.08)	(0.08)
=1 if Diagnosed Patient			179.41***	161.53***	155.15***
	–	–	(0.00)	(0.00)	(0.00)
No. of Observations	1560	558	2118	1116	2118

(a) p-value in parentheses; (b) Asterisks indicate statistical significance (*** *p* < 0.01, ** *p* < 0.05, * *p* < 0.1); (c) All figures are in local currency unit of BDT

For the matched cohort, we do not find any systematic association between WTP for corrective eyeglasses and factors such as age, gender, and marital status. However, point estimates suggest that women, older, and married respondents are willing to pay less for their vision correction through eyeglasses. We find that education plays a significant role in determining WTP. The respondents with the above primary education are willing to pay about BDT 208 (95% CI: 129–287) more compared to those with no formal education.

In terms of occupation, we find the self-employed respondents are willing to pay BDT 241 (95% CI: 67–416) more compared to the wage workers. Homemakers have significantly higher WTP (BDT 161; 95% CI: 27–349) compared to the baseline wage workers group. Interestingly, higher family size shows a negative association with WTP. However, the association is not statistically significant when the household size gets five or higher.

We further find that the respondents with higher household earnings and wealth have higher WTP for corrective eyeglasses. One standard deviation higher monthly family income is associated with a higher WTP of about BDT 119 (95% CI: 90–147). Similarly, one standard deviation higher land ownership is associated with a higher WTP of about BDT 45 (95% CI: 23–66). Families with access to mobile-phone exhibits about BDT 285 (95% CI: 148–421) higher WTP for corrective eyeglasses. We find significant anchoring effects and it is more pronounced for the diagnosed patients.

Finally, we find that a higher WTP for corrective eyeglasses are significantly associated with exposure to a health shock such as being diagnosed with refractive errors. Respondents who are diagnosed with refractive errors have higher WTP of about BDT 162 (95% CI: 104–219) which is 21.2% higher compared to the average WTP for the without-health shock group. The effects of health shock on WTP is statistically significant for all unmatched, matched, and weighted samples.

## Discussion

Refractive errors are common vision problems and corrective eyeglasses are very cost-effective for correcting refractive errors [31, 47]. However, like many other low-cost healthcare solutions, the uptake remains low [32, 48, 49]. In resource-poor settings such as Bangladesh, allocating free or subsidized health-care goods among the poor through development programs is a common practice [50, 51]. However, these strategies bring additional challenges to ensure effective coverage and prioritize those in greatest need for highly cost-effective private goods such as corrective eyeglasses. In this context, understanding the associated demand function and price mechanism through WTP studies can guide in designing an optimal pricing policy and subsidy.

Credible estimates of WTP are crucial components in designing optimal pricing policies. However, eliciting WTP through CV is essentially hypothetical in nature. More importantly, preferences can depend on the state of nature, such as being exposed to a health shock. Previous studies have associated this individual preference instability with the inability to predict future emotional reactions and the lack of experiences about the true health states [52–55]. Our paper contributes to the emerging literature on this topic. In particular, we have tested instability in consumers’ preferences in the context of the negative health shock of being diagnosed with refractive errors.

We find, by constructing two observationally equivalent groups, exposure to a negative health shock makes the with-health shock group willing to pay higher compared to the without-health shock group. The respondents diagnosed with refractive errors report higher WTP and enjoys additional consumer surplus compared to a matched population without such vision problems. Assuming that the matching protocols allow a balanced counterfactual for the patients with refractive errors, negative health shock of getting diagnosed with refractive errors appear to alter consumer preference and shifts the demand curve rightward. In other words, the stated valuation of corrective eyeglasses is higher in the state when a consumer is diagnosed with the refractive vision problem compared to a state without such health problems.

Our results are consistent with a number of economic and behavioral models of preferences. We are offering a critique of standard economic models, which are traditionally based on stable consumer preferences [18]. Our findings are rather consistent with reference-dependent preferences [56]. For example, the theory of loss aversion suggests that individuals derive utility based on their respective reference point and they value losses significantly higher compared to same sized gains [57, 58]. Prior to experiencing the negative health shock, the respondent perceives the marginal gain from a higher reference point of the healthy state. However, observationally equivalent with-health shock respondent has already incurred the utility loss and avoiding error correction can only lead to a loss of utility. So, according to a standard loss aversion model of utility, a with-health shock individual will be willing to pay more compared to a consumer belonging to a without-health shock group.

We are, however, agnostic towards invoking any particular behavioral model. We can presume other possible explanations for the observed preference instability. Firstly, individuals may not be perfectly informed about the benefits they can derive from a healthcare product. Health shock may signal about the true benefit of a health status (for example, maintaining certain visual acuity with eyeglasses), which they internalize in their benefit cost calculations once the true nature is revealed [59]. Secondly, we can also relate our findings to the theory of salience [60]. Fear of probable consequences of refractive error may cause disproportionate attention towards vision correction and corrective eyeglasses become more salient after being diagnosed as a refractive error patient. Additionally, we also find considerable within group heterogeneity in WTP for corrective eyeglasses based on observational factors such as education, income, and wealth.

As limitations of our analyses, we acknowledge that our model does not incorporate the degree of visual acuity, severity of the refractive error, and other indicators of the participant’s current health condition. This may cause further selection problem to estimate the marginal effect of health-shock on WTP for corrective eyeglasses. Moreover, we have not allowed any “opt out” option for the respondents. Hence, left censored intervals can be binding for some of the respondents. This may cause the demand curves to inflate for both with- and without-health shock groups. We also find evidence of starting point or anchoring bias suggesting the design of CV methods can also play roles in WTP inferences [39, 61].

## Conclusion

Our findings have crucial implications for both applied empirical work and for strategic marketing of healthcare products. Firstly, careful selection of the sampling frame is a prerequisite to estimating the true WTP for any healthcare product or service for the most relevant population as we find that the preferences can change after experiencing a health shock. Secondly, if we consider the with-health shock demand curve to represent the true preferences for corrective eyeglasses; subsidy can potentially generate higher marginal social welfare. Development programs can better design their initiatives based on the true demand for health care services and step ahead towards attaining self-sufficiency. Finally, differential pricing strategies can also be applied to different market segments to efficiently recover cost and increase the number of free or subsidized eyeglasses for those in greatest need.

## Supplementary information

**Additional file 1.** Comparison of alternative counterfactuals.

## Data Availability

All survey instruments, data and codes will be made available upon request.

## Abbreviations

BDT: Bangladeshi Taka
CBA: Cost Benefit Analysis
CLRM: Classical Linear Regression Model
CV: Contingent Valuation
LMIC: Low- and Middle-income Country
NGO: Non-governmental Organization
OLS: Ordinary Least Squares
PSM: Propensity Score Matching
TBDC: Triple Bounded Dichotomous Choice
WTP: Willingness to Pay
