# Supplementary material for: Health shock and preference instability: assessing health-state dependency of willingness-to-pay for corrective eyeglasses
Source: Health Econ Rev. 2019 Nov 7;9:32. doi: 10.1186/s13561-019-0249-3 (PMC6836482; doi:10.1186/s13561-019-0249-3)
Supplement: Supplementary file 1 — Additional file 1. Comparison of alternative counterfactuals. [file 13561_2019_249_MOESM1_ESM.docx]

**Health Shock and Preference Instability: Assessing Health-State Dependency of Willingness-to-Pay for Corrective Eyeglasses**

Muhammed Nazmul Islam^1^, Atonu Rabbani^1,2, *^, Malabika Sarker^1,3^

^1^BRAC James P Grant School of Public Health, BRAC University

68 Shahid Tajuddin Ahmed Avenue, Mohakhali, Dhaka–1212, Bangladesh

^2^Department of Economics, University of Dhaka

Dhaka 1000, Bangladesh

^3^Institute of Public Health, Heidelberg University

Im Neuenheimer Feld 130.3, 69120 Heidelberg, Germany

*Corresponding Author:

Atonu Rabbani

Associate Professor

Department of Economics

University of Dhaka

Dhaka 1000, Bangladesh

&

Associate Scientist

James P Grant School of Public Health

BRAC University

Email: atonu.rabbani@du.ac.bd

Phone: +8801730441787

**Additional File 1**

**Table A1: Balance-Checking Criteria**

| **Variables** | **1 to 1 Matching (Replaced)** | **1 to 1 Matching (Not Replaced)** | **Kernel Matching** | **Radius Matching** | **Mahalanobis Metric Matching** |
| --- | --- | --- | --- | --- | --- |
| **Criterion 2 : Mean Difference as Percentage of Average Standard Deviation** | | | | | |
| Female | 1.801 | 19.265 | 21.556 | 17.815 | 4.598 |
| Married | 12.307 | 12.292 | 9.535 | 27.693 | 3.056 |
| Owns a TV | 4.671 | 1.022 | 1.266 | 4.997 | 4.143 |
| Owns a mobile | 8.968 | 4.478 | 0.000 | 22.109 | 0.518 |
| Contribution to Income | 1.364 | 8.390 | 9.038 | 5.143 | 5.159 |
| Family Income | 6.552 | 18.232 | 17.991 | 26.855 | 19.219 |
| Age | 13.596 | 0.628 | 4.422 | 19.307 | 17.140 |
| Respondent’s Income | 2.933 | 11.520 | 12.508 | 8.436 | 7.361 |
| Family Size: |  |  |  |  |  |
| 1 - 2 | 0.967 | 0.000 | 3.802 | 11.233 | 10.838 |
| 3 - 4 | 11.219 | 1.009 | 7.924 | 10.841 | 17.266 |
| 5+ | 10.408 | 1.001 | 5.403 | 18.515 | 11.100 |
| Occupation: |  |  |  |  |  |
| Wage Workers | 3.965 | 1.745 | 19.148 | 26.937 | 0.590 |
| Self-employed | 0.652 | 4.150 | 6.594 | 12.191 | 1.656 |
| Garment Workers | 1.116 | 1.697 | 5.325 | 18.403 | 2.869 |
| Service | 0.000 | 13.520 | 11.599 | 26.918 | 0.493 |
| Homemakers | 2.335 | 21.237 | 23.683 | 13.692 | 0.422 |
| Others | 0.880 | 14.699 | 13.496 | 26.560 | 2.195 |
| Education: |  |  |  |  |  |
| Never been to School | 16.672 | 14.952 | 14.517 | 18.826 | 0.924 |
| Primary | 6.768 | 7.636 | 7.139 | 26.635 | 0.000 |
| Secondary | 3.089 | 12.889 | 14.420 | 19.391 | 0.569 |
| SSC/Dakhil/Equivalent | 0.855 | 2.006 | 1.429 | 17.929 | 1.407 |
| HSC/Fazil/Equivalent | 8.697 | 12.554 | 13.668 | 27.296 | 1.542 |
| Graduate | 5.972 | 13.524 | 13.524 | 30.528 | 0.716 |
| Post Graduate | 4.680 | 19.080 | 19.080 | 27.511 | 2.011 |
| Others | 3.267 | 30.926 | 31.785 | 30.926 | 0.000 |
| **Criterion 3: Percent Bias Reduced in Means of Explanatory Variables** | | | | | |
| Female | 100.9 | 109.5 | 110.6 | 108.8 | 97.7 |
| Married | 94.2 | 105.5 | 104.3 | 111.8 | 101.4 |
| Owns a TV | 101.8 | 100.4 | 99.5 | 98.0 | 101.6 |
| Owns a mobile | 98.1 | 99.1 | 100.0 | 94.7 | 99.9 |
| Contribution to Income | 100.6 | 96.4 | 96.1 | 102.2 | 97.8 |
| Family Income | 133200.0 | 347180.0 | 341740.0 | 501150.0 | 357100.0 |
| Age | 330.0 | 110.0 | 170.0 | 190.0 | 160.0 |
| Respondent’s Income | 32440.0 | 112380.0 | 121920.0 | 79360.0 | 66790.0 |
| Family Size: |  |  |  |  |  |
| 1 - 2 | 100.3 | 100.0 | 101.2 | 103.7 | 96.9 |
| 3 - 4 | 94.5 | 99.5 | 96.1 | 105.4 | 108.6 |
| 5+ | 105.2 | 100.5 | 102.7 | 90.9 | 94.5 |
| Occupation: |  |  |  |  |  |
| Wage Workers | 100.7 | 100.3 | 97.7 | 106.1 | 100.1 |
| Self-employed | 100.2 | 101.3 | 102.1 | 104.0 | 99.5 |
| Garment Workers | 99.8 | 99.7 | 99.1 | 104.1 | 99.5 |
| Service | 100.0 | 94.8 | 95.5 | 90.3 | 99.8 |
| Homemakers | 98.9 | 110.3 | 111.5 | 106.6 | 100.2 |
| Others | 100.4 | 93.6 | 94.1 | 88.9 | 101.0 |
| Education: |  |  |  |  |  |
| Never been to School | 107.5 | 106.7 | 106.5 | 108.5 | 100.4 |
| Primary | 97.5 | 103.0 | 102.8 | 111.1 | 100.0 |
| Secondary | 101.1 | 104.8 | 105.4 | 107.4 | 100.2 |
| SSC/Dakhil/Equivalent | 99.7 | 99.3 | 99.5 | 94.3 | 100.5 |
| HSC/Fazil/Equivalent | 97.3 | 96.2 | 95.9 | 92.6 | 99.5 |
| Graduate | 98.4 | 96.6 | 96.6 | 93.5 | 99.8 |
| Post Graduate | 99.1 | 97.0 | 97.0 | 96.2 | 99.6 |
| Others | 99.4 | 96.40 | 96.3 | 96.4 | 100.0 |
| **Criterion 4: Comparison of Treatment and Control Density Estimates - Kalmogorov-Smirnov (K-S) Test** | | | | | |
| Female | 0.999 | 0.013 | 0.004 | 0.027 | 0.998 |
| Married | 0.318 | 0.355 | 0.680 | 0.001 | 0.999 |
| Owns a TV | 0.999 | 0.999 | 0.999 | 0.999 | 0.999 |
| Owns a mobile | 0.999 | 0.999 | 0.999 | 0.395 | 0.999 |
| Contribution to Income | 0.903 | 0.580 | 0.222 | 0.085 | 0.251 |
| Family Income | 0.013 | 0.000 | 0.000 | 0.000 | 0.000 |
| Age | 0.009 | 0.009 | 0.009 | 0.000 | 0.001 |
| Respondent’s Income | 0.995 | 0.395 | 0.283 | 0.073 | 0.531 |
| Family Size: |  |  |  |  |  |
| 1 - 2 | 0.999 | 0.999 | 0.999 | 0.824 | 0.958 |
| 3 - 4 | 0.355 | 0.999 | 0.779 | 0.395 | 0.032 |
| 5+ | 0.438 | 0.999 | 0.988 | 0.019 | 0.355 |
| Occupation: |  |  |  |  |  |
| Wage Workers | 0.999 | 0.999 | 0.998 | 0.251 | 0.999 |
| Self-employed | 0.999 | 0.999 | 1.000 | 0.779 | 0.999 |
| Garment Workers | 0.999 | 0.999 | 0.999 | 0.730 | 0.999 |
| Service | 0.999 | 0.438 | 0.630 | 0.011 | 0.999 |
| Homemakers | 0.999 | 0.005 | 0.001 | 0.172 | 0.999 |
| Others | 0.999 | 0.196 | 0.283 | 0.002 | 0.999 |
| Education: |  |  |  |  |  |
| Never been to School | 0.085 | 0.172 | 0.196 | 0.038 | 0.999 |
| Primary | 0.995 | 0.958 | 0.976 | 0.002 | 0.999 |
| Secondary | 0.999 | 0.531 | 0.395 | 0.098 | 0.999 |
| SSC/Dakhil/Equivalent | 0.999 | 0.999 | 0.999 | 0.318 | 0.999 |
| HSC/Fazil/Equivalent | 0.988 | 0.824 | 0.730 | 0.098 | 0.999 |
| Graduate | 0.999 | 0.903 | 0.903 | 0.196 | 0.999 |
| Post Graduate | 0.999 | 0.958 | 0.958 | 0.824 | 0.999 |
| Others | 0.999 | 0.866 | 0.866 | 0.866 | 0.999 |
| **Criterion 5: Comparing Density of Propensity Scores** | | | | | |
| P value of Combined K-S Test | 0.995 | 0.000 | 0.000 | 0.000 | – |
|  |  |  |  |  |  |

Note: (a) Criterion 1 is to find a cohort with insignificant mean differences between the explanatory variables of with- and without-health-shock groups which is shown in table 2, (b) criterion 2 is to find a cohort with low mean difference as a percentage of the average standard deviation, (c) criterion 3 is to find a cohort with 100 percent bias reduction in the means of explanatory variables, (d) criterion 4 is to find a cohort where the differences between the density estimates of the explanatory variables of with- and without-health-shock groups are insignificant, (e) criterion 5 is to find a cohort with insignificant differences in the density estimates for the propensity scores of the two groups.

Table A2: Summary Statistics for Sub-samples in Other Matching Methods

|  | **[1]** | **[2]** | **[3]** | **[4]** | **[5]** | **[6]** |
| --- | --- | --- | --- | --- | --- | --- |
|  | **Kernel Matching**  [N = 558] | ***P* Value** | **Radius Matching**  [N = 558] | ***P* Value** | **Mahalanobis Metric Matching**  [N = 558] | ***P* Value** |
| Female | 62.9 % | 0.000 *** | 61.1 % | 0.003 *** | 50.0 % | 0.437 |
| Married | 73.7 % | 0.112 | 81.2 % | 0.000 *** | 70.8 % | 0.601 |
| Owns a TV | 80.5 % | 0.820 | 79.0 % | 0.411 | 82.6 % | 0.485 |
| Owns a mobile | 96.2 % | 0.999 | 90.9 % | 0.000 *** | 96.1 % | 0.877 |
| Respondent's Contribution to Family Income | 30.7 % | 0.126 | 36.8 % | 0.388 | 32.4 % | 0.377 |
| Family Income (BDT) | 17,863.3 | 0.004 *** | 16,269.2 | 0.000 *** | 17,709.7 | 0.002 *** |
| Land (Decimal) | 19.14 | 0.000 *** | 35.7 | 0.001 *** | 36.0 | 0.004 *** |
| Age | 39.3 | 0.472 | 5,486.0 | 0.160 | 5,611.7 | 0.221 |
| Respondent's Income (BDT) | 5,060.4 | 0.037 ** | 12.72 | 0.000 *** | 29.37 | 0.001 *** |
| **Family Size** |  |  |  |  |  |  |
| 1-2 | 11.8 % | 0.392 | 14.3 % | 0.005 *** | 7.5 % | 0.010 ** |
| 3-4 | 39.1 % |  | 48.4 % |  | 51.6 % |  |
| 5+ | 49.1 % |  | 37.3 % |  | 40.9 % |  |
| **Occupations** |  |  |  |  |  |  |
| Wage Workers | 0.6 % | 0.000 *** | 9.0 % | 0.000 *** | 3.0 % | 0.994 |
| Self-employed | 12.5 % |  | 14.4 % |  | 9.9 % |  |
| Garment Workers | 2.5 % |  | 7.5 % |  | 2.9 % |  |
| Service | 16.3 % |  | 11.1 % |  | 20.6 % |  |
| Homemakers | 45.2 % |  | 40.3 % |  | 33.9 % |  |
| Others | 22.9 % |  | 17.7 % |  | 29.8 % |  |
| **Education** |  |  |  |  |  |  |
| Never been to School | 31.2 % | 0.000 *** | 33.2 % | 0.000 *** | 25.1 % | 0.999 |
| Primary | 20.4 % |  | 28.7 % |  | 17.6 % |  |
| Secondary | 19.7 % |  | 21.7 % |  | 14.5 % |  |
| SSC/Dakhil/Equivalent | 14.0 % |  | 8.8 % |  | 15.0 % |  |
| HSC/Fazil/Equivalent | 8.1 % |  | 4.8 % |  | 11.7 % |  |
| Graduate | 5.2 % |  | 2.1 % |  | 8.4 % |  |
| Post Graduate | 1.3 % |  | 0.5 % |  | 3.9 % |  |
| Others | 0.1 % |  | 0.2 % |  | 3.8 % |  |

Notes: (a) p-value for comparison between With-Health-Shock group and Matched sub-samples of Without-Health-Shock group: using t-test of difference in means for continuous variables or Chi-square test of independence for categorical variables; (b) Asterisks indicate statistical significance (*** p <0.01, ** p <0.05, * p <0.1), (a) US$ 1 is equivalent to BDT 84.397.
